# An Overview of Polymer Composite Films for Antibacterial Display Coatings and Sensor Applications

**DOI:** 10.3390/polym15183791

**Published:** 2023-09-17

**Authors:** Swathi Ippili, Jang-Su Jung, Alphi Maria Thomas, Van-Hoang Vuong, Jeong-Min Lee, Mizaj Shabil Sha, Kishor Kumar Sadasivuni, Venkatraju Jella, Soon-Gil Yoon

**Affiliations:** 1 Department of Materials Science and Engineering, Chungnam National University, Daejeon 34134, Republic of Korea; 2jsooya@gmail.com (J.-S.J.); alphirosary@gmail.com (A.M.T.); hoangvv.cnu@gmail.com (V.-H.V.); minimi592@gmail.com (J.-M.L.); 2 Center for Advanced Materials, Qatar University, Doha P.O. Box 2713, Qatar; misajmisfa@gmail.com (M.S.S.); kishor_kumars@yahoo.com (K.K.S.); 3 Department of Mechanical and Industrial Engineering, Qatar University, Doha P.O. Box 2713, Qatar

**Keywords:** polymer composite, antibacterial, display coating, sensor, multifunctional

## Abstract

The escalating presence of pathogenic microbes has spurred a heightened interest in antimicrobial polymer composites tailored for hygiene applications. These innovative composites ingeniously incorporate potent antimicrobial agents such as metals, metal oxides, and carbon derivatives. This integration equips them with the unique ability to offer robust and persistent protection against a diverse array of pathogens. By effectively countering the challenges posed by microbial contamination, these pioneering composites hold the potential to create safer environments and contribute to the advancement of public health on a substantial scale. This review discusses the recent progress of antibacterial polymer composite films with the inclusion of metals, metal oxides, and carbon derivatives, highlighting their antimicrobial activity against various pathogenic microorganisms. Furthermore, the review summarizes the recent developments in antibacterial polymer composites for display coatings, sensors, and multifunctional applications. Through a comprehensive examination of various research studies, this review aims to provide valuable insights into the design, performance, and real-time applications of these smart antimicrobial coatings for interactive devices, thus enhancing their overall user experience and safety. It concludes with an outlook on the future perspectives and challenges of antimicrobial polymer composites and their potential applications across diverse fields.

## 1. Introduction

The presence of harmful microorganisms in the surrounding environment can give rise to a wide range of social, economic, and human issues [1]. Indeed, the contamination of material surfaces by microbes plays a significant role in the rapid transmission of infectious diseases among individuals [2]. Interactive displays utilizing touchscreen technology have become prevalent in healthcare, public spaces, and industries, allowing easy information access through touch interactions in both public and semi-public areas [3,4]. However, these touchscreens serve as sources of various harmful pathogens, including bacteria, viruses, fungi, and parasites, which can readily spread to humans through the operation of such devices [5,6]. Specifically, the recent COVID-19 pandemic has heightened concerns regarding the use of touchscreen devices in public spaces, as they can be touched by multiple individuals, potentially facilitating the transmission of harmful pathogens and posing an infection risk [7,8]. A crucial strategy in disease prevention involves the regular cleaning and frequent disinfection of touchscreens. Nevertheless, conventional disinfection methods, which rely on chemicals like ethanol, isopropanol, and hypochlorite, are unsuitable for disinfecting touchscreens due to their sensitivity to these substances [9]. In addition to touchscreen devices, other surfaces, like door handles, elevator buttons, and escalator rails, in public areas can also become contaminated and pose a potential risk of the spread of microbial pathogens [10].

Furthermore, the rapid advancement of technology and the growing influence of the Internet of Things (IoT) have greatly enabled the integration of flexible wearable electronic devices in various domains, including personal healthcare, electronic skin systems, and haptic sensors [11,12,13]. These devices provide innovative health monitoring, improved human–computer interactions, and seamless technology integration into daily life by adhering to the skin and tracking data regarding pulse rate, physiological condition, body temperature, and blood glucose levels [14]. Extended skin contact with these devices can promote harmful pathogen growth, thereby increasing health risks. Proper hygiene is crucial even with chemical disinfection, as frequent contact can lead to recontamination. Antibacterial surface coatings offer an innovative way to protect interactive displays, wearables, and other surfaces from bacterial contamination [15]. A diverse array of nanomaterials fall under scrutiny, encompassing metals (such as silver, copper, and zinc), metal oxides (like zinc oxide, silver oxide, and copper oxide), and carbon-based materials. These materials are extensively researched due to their potential to exhibit antibacterial properties, thereby combatting a wide spectrum of microorganisms, including bacteria, fungi, and viruses [16,17,18]. Nevertheless, attaining substantial transparency through the deposition of these films via the solution process poses a significant challenge due to the tendency of nanoparticles to aggregate [19]. Furthermore, the mechanical properties of films based on solution-processed nanoparticles tend to be subpar, mainly because of weak adhesion to the surface. Moreover, as a novel material category, polymer composites consisting of the integration of antibacterial nanomaterials have garnered significant attention as a means of applying antibacterial coatings [20]. Polymer composite coatings present the advantage of exceptional mechanical resilience and hydrophobic characteristics [21]. The hydrophobic property of surface coatings assumes a critical role in impeding the proliferation and dissemination of microorganisms. Moreover, recent research has centered on these antibacterial polymer composite films for their application in a range of sensors, encompassing touch, pressure, humidity, and temperature sensors [22,23].

**Figure 1:**
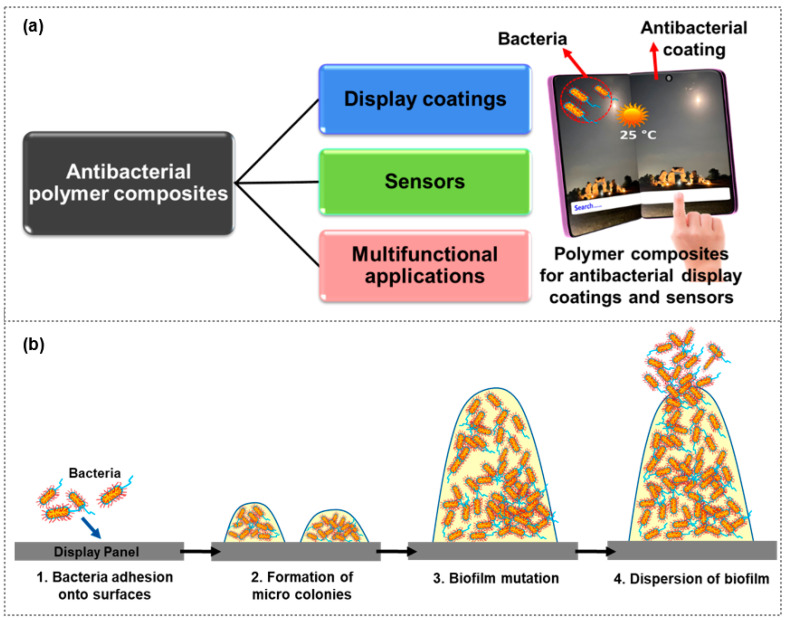
(**a**) Conceptual illustration of antibacterial polymer composites for display coatings, sensor, and multifunctional applications, and (**b**) bacterial colonization on interactive displays.

This comprehensive review delves into the recent progress made in the field of polymer composite films, specifically highlighting their applications in two key areas: display coatings and sensors (Figure 1a). Nonetheless, touchscreen surfaces can serve as breeding grounds for various microorganisms, including bacteria, fungi, and algae, as visualized in Figure 1b. By delving into the latest developments, this review aims to provide a detailed understanding of how polymer composite films are being harnessed to enhance the performance and functionality of displays and sensors along with the antibacterial activity. Through a thorough examination of various studies and innovations, this review sheds light on the evolving landscape of polymer composites in these domains, elucidating their potential to reshape the way we approach display technology and sensor applications.

## 2. Antibacterial Polymer Composite Materials

Commonly, polymers lack intrinsic antibacterial capabilities, necessitating innovative approaches to bestow them with antimicrobial properties. To this end, researchers have fervently explored avenues of modification and functionalization, seeking to augment polymers’ ability to combat microbial threats. A particularly notable strategy involves the infusion of antimicrobial additives such as metals, metal oxides, and carbon-based materials into polymer matrices. This integration, while altering the composition, serves as a catalyst for bestowing antibacterial activity upon the resultant polymer composites. This methodology has garnered substantial interest across diverse scientific domains, owing to its potential to elevate the antimicrobial efficacy of polymers, thereby addressing crucial concerns associated with microbial contamination in various applications.

### 2.1. Metals-Incorporated Polymer Composites

Polymer composite materials infused with metals showcase antibacterial efficacy owing to the antimicrobial attributes of metallic nanoparticles. These nanoparticles can efficiently exterminate and impede the proliferation of bacteria on the surface of the composite material. The pivotal advantage of integrating these materials into composite coatings lies in their capability to deter the attachment of microbial pathogens while augmenting their antimicrobial potency [24]. Furthermore, polymers can enhance the mechanical attributes of composite films, which are crucial for display and sensor coating applications (Figure 1a). Multiple studies have showcased the antibacterial effectiveness of polymer composites achieved through the integration of diverse metallic nanoparticles, encompassing silver, gold, copper, and zinc [25,26,27]. The antimicrobial efficacy of nanomaterials is intricately linked to their structural and physical characteristics, including size, shape, chemical composition, surface area, and zeta potential. The antimicrobial actions of metals encompass a range of processes, such as producing reactive oxygen species, releasing cations, inducing biomolecule harm, depleting ATP, and interacting with cell membranes, all of which contribute to the eradication of bacteria [28]. Silver is commonly chosen as one of the metals to be infused into a polymer matrix, thereby imparting the polymer with antimicrobial capabilities. Silver stands out as one of the most frequently employed metals for integration into polymer matrices to achieve antimicrobial effects [25]. For example, Hoque et al. demonstrated the dual function of polymer–silver nanocomposite with excellent antimicrobial activity against various bacteria and fungi. The preparation of the silver nanocomposites involved the biodegradable polymer N, N-dimethyl-N-hexadecyl ammonium chitin tosylate (Q-DMHC48) [29]. The surfaces coated with the nanocomposite demonstrated excellent antimicrobial activity in various drug-resistant bacteria, including methicillin-resistant *Staphylococcus aureus* (MRSA), vancomycin-resistant *Enterococcus faecium* (VRE) *P. aeruginosa*, and *Klebsiella pneumoniae*, as well as pathogenic fungi such as *Candida* spp. and *Cryptococcus* spp. In addition, nanocomposite-coated surfaces exhibited rapid killing and long-lasting antimicrobial activity, maintaining effectiveness over an extended duration. Moreover, when applied to catheters, the nanocomposites effectively reduced the burden on the catheter and in the tissues surrounding it in a mice model.

**Figure 2:**
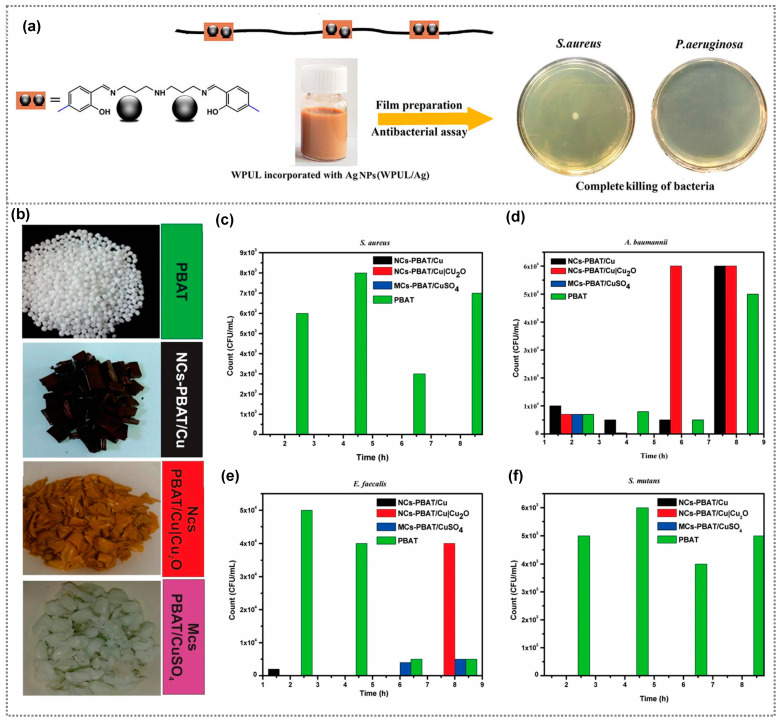
(**a**) Formation of a Ag/WPUL nanocomposite through the coordination of silver ions with WPUL and its antibacterial activity against *S. aureus* and *P. aeruginosa*. (**a**) Reprinted from Ref. [32]. Copyright 2020, Elsevier. (**b**) Captured images of PBAT, PBAT/Cu, PBAT/Cu|Cu_2_O, and PBAT/CuSO_4_, showing antibacterial activity and colonization count at a 3% concentration against (**c**) *S. aureus*, (**d**) *A. baumanni*, (**e**) *E. faecalis*, and (**f**) *S. mutan*, respectively. (**b**–**f**) Reprinted from Ref. [40]. Copyright 2019, Springer.

Several studies have also focused on the preparation of antibacterial polyurethane coatings impregnated with the incorporation of Ag nanoparticles, which are capable of releasing bactericidal silver ions upon contact with bacteria and fungi [30,31]. However, these coatings are generally effective only for short-term applications due to the high diffusivity of Ag NPs and their tendency to aggregate. Thus, Mohammadi et al. prepared a silver(I) complex with a Schiff base ligand (SBL)-extended waterborne polyurethane (WPUL/Ag) with high storage stability and a low aggregation tendency [32]. The WPUL/Ag composite coating exhibited strong antibacterial activity against both Gram-positive Staphylococcus aureus and Gram-negative *Pseudomonas aeruginosa* bacteria, achieving a bacterial reduction of 99.99%, while the WPUL revealed no antibacterial activity (Figure 2a). Copper is inexpensive compared with other metals, and is a widely used material for preparing various antibacterial polymer composites [33]. For example, Pinto et al. prepared a cellulose-based biopolymer nanocomposite using copper nanostructures, namely nanoparticles and nanowires, and investigated its antibacterial efficiency against *S. aureus* and *K. pneumoniae* [34]. The composite with copper nanowires exhibited less antibacterial activity than the nanoparticle-based composite. Additionally, a significant improvement in antibacterial activity was observed with increasing copper content. In another study, antibacterial thermoplastic polyurethane (TPU) was prepared by incorporating 1 wt% copper particles through the melt blending method [35]. They observed the resulting composite films successfully hindered the growth of Staphylococcus aureus (*S. aureus*) and *Escherichia coli* (*E. coli*), effectively inhibiting biofilm formation. Furthermore, Maximino et al. created antimicrobial polypropylene composites utilizing copper nanoparticles functionalized with polyethyleneimine and 4-aminobutyric acid [36]. These composites were prepared using various concentrations of copper nanoparticles ranging from 0.25%, 1%, 2.5%, and 5% by weight, and their antibacterial activity toward P. aeruginosa and *S. aureus* was investigated. Notably, the 5 wt% copper–polymer nanocomposite demonstrated robust antibacterial activity compared to the other concentrations. It achieved 100% antibacterial activity within 2 h against *P. aeruginosa,* and within 4 h against *S. aureus*. Gold nanoparticles are extensively employed in various biomedical applications due to their strong stability and excellent biocompatibility [37]. They are also readily modifiable, allowing for easy customization, and their antibacterial properties can be further enhanced by altering their structure, and size, or incorporating additional ingredients. As an example, Futyra et al. developed gold–chitosan nanocomposite films and investigated their antibacterial activity against strains of *S. aureus* and *P. aeruginosa* [38]. Chitosan, a biocompatible and biodegradable polymer, was used as a reducing and stabilizing agent in the synthesis of gold nanoparticles. The resultant nanocomposite films displayed potent antibacterial properties with minimal cytotoxicity. Additionally, Zaporojtchenko et al. prepared antibacterial composite coatings composed of Ag–Au/polytetrafluorethylene (PTFE) through the co-sputtering of Ag and PTFE, where a small amount of Au (~0.1 nm) was deposited on the surface of Ag/PTFE composite films [39]. The resultant composite displayed a greater antibacterial effect than the Ag/PTFE films against *S. aureus* and *S. epidermidis*. Indeed, the concentration of nanoparticles (NPs) plays a pivotal role in toxicity, with higher concentrations leading to increased ion release. It is important to note that employing higher concentrations of metal nanoparticles might compromise the transparency of composite films.

### 2.2. Metal Oxide-Incorporated Polymer Composites

Metal oxides are substances formed when a metal reacts with oxygen. They have a wide range of properties and applications in various fields. Specifically, in the context of antimicrobial action, numerous metal oxide nanoparticles such as ZnO, CuO, MgO, SnO_2_, TiO_2_, and Fe_2_O_3_ have been investigated for their ability to counteract a broad spectrum of harmful microorganisms due to their robust durability, enduring stability, and minimal toxicity [41]. Nanocomposites directly interact with bacterial cell membranes through the electrostatic interactions between released ions and the bacterial cell wall, or the release of heavy metal ions due to surface oxidation, which causes disruption of the cell membrane, leading to bacterial damage [42,43]. Metal oxide nanoparticles, particularly those like ZnO and TiO_2_, can produce reactive oxygen species (ROS) upon exposure to light or other stimuli. These ROS, such as hydrogen peroxide, singlet oxygen, superoxide anions, and hydroxyl radicals, can damage bacterial cell components like DNA, proteins, and lipids, ultimately leading to cell death [41,42]. Several studies have also demonstrated the antibacterial activity of metal oxide–polymer nanocomposite coatings in various applications [43]. Among various metal oxides, ZnO stands out as a highly promising material due to its bio-safe nature, biocompatibility, and cost-effectiveness. Its antimicrobial properties have been thoroughly investigated, showcasing effectiveness against a diverse array of pathogenic organisms [44]. For example, Dimitrakellis et al. investigated the activity of ZnO/polymethyl(methacrylate) (PMMA) nanocomposite films prepared through a solution process, both with and without atmospheric plasma treatment, against *E. coli* [45]. Plasma-treated composite films exhibited a significant enhancement in antibacterial activity due to the gradual exposure and aggregation of ZnO nanoparticles on the nanocomposite surface after plasma etching. However, nanoparticles prepared using a solution method often result in the agglomeration of the nanoparticles, which not only affects the antibacterial activity but also reduces the transmittance of the composite coatings. In contrast, metal oxide thin films prepared by sputtering exhibit excellent antibacterial activity and mechanical durability [46]. For example, ZnO-PTFE composite films prepared by the sputtering method exhibited excellent antibacterial activity and have been used for display coating applications. In addition, composite films displayed a hydrophobic nature compared with ZnO films. Copper oxide nanoparticles have also garnered significant attention for their remarkable antibacterial activity against a wide spectrum of bacteria. Haider et al. prepared Poly(lactide-co-glycolide) (PLGA)/CuO composite nanofibers by electrospinning [47]. They investigated the antimicrobial activity against *E. coli* and *S. aureus* bacterial strains, and the composite films notably inhibited the growth of both bacteria. In another report, a comparative study of the antibacterial effect of poly(butylene adipate-co-terephthalate) (PBAT)-based nanocomposites synthesized using copper nanoparticles, copper/cuprous oxide (Cu/Cu_2_O) nanoparticles, and copper sulfate (CuSO_4_) was carried out against *S. aureus*, *Acinetobacter baumanni*, *Enterococcus faecalis*, *Streptococcus mutans* [40]. Antimicrobial assessments demonstrated that the nanocomposite with Cu/Cu_2_O nanoparticles resulted in antibacterial activity against *E. faecalis* and *S. mutans*, coupled and excellent bactericidal effects against *S. aureus* (Figure 2b–f). Meanwhile, the composite with CuSO_4_ exhibited effective bactericidal responses against *A. baumannii*, *E. faecalis*, and *S. mutans*, and displayed excellent efficacy against *S. aureus*. In contrast, PBAT without additives did not exhibit bactericidal properties upon contact with the bacterial strains. Furthermore, nanocomposite materials containing NiO and MgO combined with chitosan biopolymer have demonstrated antibacterial activity against both *E. coli* and *S. aureus* bacterial strains [48].

### 2.3. Carbon Derivates-Incorporated Polymer Composites

Carbon-based nanomaterials are emerging as promising platforms with diverse applications due to their unique mechanical, electronic, and biological properties. Notably, carbon nanostructures such as diamond-like carbon (DLC), graphene, graphene oxide, carbon nanotubes (CNTs), and fullerene have garnered interest attention for their potent antibacterial properties and their ability to combat a broad spectrum of pathogens [49,50]. Carbon nanomaterials exhibit an antibacterial effect through the physical disruption of cell membranes, generation of reactive oxygen species, photothermal/photocatalytic effect, inhibition of cell adhesion, electrostatic interactions, and intracellular disruption [49,51]. Indeed, recent research has highlighted the antibacterial potential of polymer composites integrated with carbon-based nanomaterials [52].

**Figure 3:**
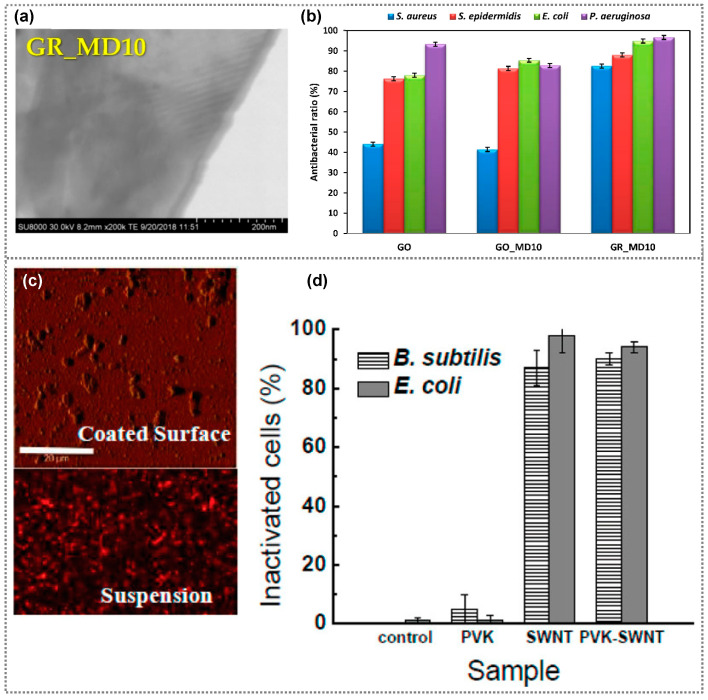
(**a**) FESEM image of GR functionalized with MD10 (GR-MD10), and (**b**) Antibacterial ratio of GO, GO-MD10, and GR-MD10. (**a**,**b**) Reprinted from Ref. [54]. (**c**) Atomic microscopic and fluorescence microscopic images of biofilm formation of *E. coli* on PVK-SWNT-coated and suspension surfaces, respectively; and (**d**) Correlation percentage of nonviable *E. coli* and *B. subtilis* cells (inactivated cells %) after interaction with PVK, SWNT (1 mg/mL), and PVK-SWNT. (**c**,**d**) Reprinted from Ref. [56]. Copyright 2011, American Chemical Society.

In a specific case, Santos et al. prepared an antibacterial polymer nanocomposite composed of polyvinyl N-carbazole (PVK) and graphene oxide (GO), and investigated its antibacterial properties against *E. coli* [53]. The prepared composite films exhibited improved antimicrobial activity compared with compared to both the unmodified surface and a surface modified solely with pure GO. In addition, Placha et al. investigated antibacterial activity against *S. aureus*, *E. coli*, *S. epidermidis*, and *P. aeruginosa* on functionalized graphene oxide and graphene using the quaternized statistical copolymer P(MTA90-co-DOMA10), MD10 (Figure 3a,b) [54]. The introduction of MD10 enhances the antibacterial effects of graphene oxide (GO) against most bacteria, except for *P. aeruginosa* and *S. aureus*. Notably, functionalized GR with MD10 exhibits the most favorable outcomes, possibly due to its increased positive charge, contributing to improved efficacy. Another study examined ultra-thin fibers manufactured from poly(methyl methacrylate) and graphene nanoplatelets (GNPs) at different concentrations (2%, 4%, and 8%) for their potential antibacterial uses against *E. coli* and *P. aeruginosa* [55]. The quantity of GNPs in composite films played a vital role in influencing bacterial growth. Surprisingly, the findings demonstrated that fibers containing 2% and 4% GNPs promoted microbial growth, while fibers with 8% GNPs displayed antimicrobial properties. Moreover, the antimicrobial activity of a polymer nanocomposite comprising 97 wt% polyvinyl-N-carbazole (PVK) and 3 wt% single-walled carbon nanotubes (SWNT) was investigated in both water suspensions, and as thin film coatings [56]. The toxic effects of different concentrations of this PVK-SWNT nanocomposite were tested against planktonic cells and biofilms of *E. coli* and *B. subtilis*. The results indicated that the PVK-SWNT nanocomposite exhibited antibacterial activity at all concentration levels. In particular, PVK-SWNT with a concentration of 1 mg/mL exhibited superior bacterial damage of 94% for *E. coli* and 90% for *B. subtilis* in planktonic cells (Figure 3c,d). The antibacterial activity of several polymer composite materials against various microorganisms discussed in this review article is summarized in Table 1.

## 3. Antibacterial Polymer Composites for Display Coating Applications

Displays play a pivotal role in conveying information and presenting visual data across diverse media platforms. Interactive touchscreen devices, allowing users to engage with onscreen content through digital touch interactions, are ubiquitous worldwide [57,58]. However, various microorganisms such as bacteria, fungi, and algae thrive and mutate on touchscreen surfaces, as depicted in Figure 1b, potentially causing the spread of infectious diseases to humans [59,60,61]. Moreover, these microorganisms’ metabolism can produce chemical byproducts that corrode the touchscreen, diminishing its sensitivity [62]. Additionally, bacterial colonies affect the touchscreen’s optical properties and can irreversibly damage it [63]. Hence, antibacterial technology can effectively curb the proliferation of these bacteria, curbing the spread of severe contagions [64,65,66]. A hydrophobic coating material is advantageous as it reduces microorganisms’ adhesion to the display, thus thwarting gene mutation and biofilm formation [67,68]. Furthermore, this hydrophobic attribute offers anti-fingerprinting functionality when applied to touchscreens. Moreover, high transmittance, antireflective capabilities, and robust mechanical durability are prerequisites for touchscreen displays [69,70]. Thus, there is significant interest in developing antimicrobial coating materials that encompass additional traits like transmittance, hydrophobicity, and mechanical properties. These antibacterial coatings must combine desirable attributes, including robust antibacterial efficacy, environmental safety, low toxicity, cost-effectiveness, and ease of fabrication [71,72,73,74]. Numerous studies have explored various compounds, such as metals and metal oxides, possessing the aforementioned attributes, and utilized them as surface coatings.

**Figure 4:**
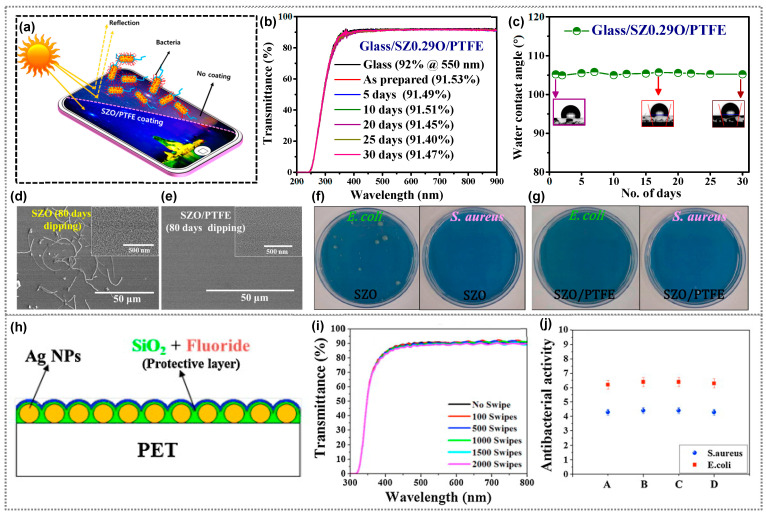
(**a**) Schematic illustration of SZO/PTFE-based transparent, antireflective, and antibacterial composite coatings for display panel application, (**b**) Transmittance, (**c**) Water contact angles of SZO/PTFE composite coatings after exposing to ambient air over a period of days, SEM images of (**d**) SZO and (**e**) SZO/PTFE composite films after 80 days of dipping into DI water, and antibacterial activity of (**f**) SZO and (**g**) SZO/PTFE coatings against *E. coli*, and *S. aureus*. (**a**–**g**) Reprinted from Ref. [62]. Copyright 2022, American Chemical Society. (**h**) Schematic illustration of nanocomposite coatings composed of a protective layer of PTFE/SiO_2_ and silver nanoparticles, (**i**) Transmittance of composite coatings with respective number of swipes, and (**j**) Antibacterial activity of composite coatings for (A) without swiping, (B) after 2000 touches, (C) after 2000 swipes, and (D) after bending at 3 cm from the original state for 30 s. (**h**–**j**) Reprinted from Ref. [75]. Copyright 2015, Elsevier.

For smartphone panel applications, Choi et al. used radio-frequency sputtering to create antibacterial ZnO thin films [71]. These films, annealed at 100 °C, had high transmittance (~91.3%) and hydrophobicity (angle ~109° ± 2°). They effectively fought *E. coli* and S. aureus, akin to Ag and Zn nanoparticles. Enhanced by increased roughness, annealed ZnO films excelled over the as-prepared samples. Despite 2000 touches, they maintained mechanical robustness with a minor (~3%) transmittance drop, displaying resistance to fingerprints and hydrophobic traits, making them promising coatings. The sputtered ZnAl_2_O_4_ thin film prepared by the same research group exhibits enhanced properties compared to neat ZnO thin films due to aluminum incorporation [74]. This incorporation (85 at.% Al) improves resistance against DI water and acidic water, restricting the release of Zn^2+^ and Al^3+^ ions even after extended immersion (over 10 days). Consistently, the transmittance and morphology remain unchanged after 10 days, yet the high water contact angles result from thermal treatment-induced surface modification, potentially leading to sporadic properties upon long-term use [64]. As an alternative, thin polymer passivation significantly enhances water resistance in antibacterial films while leaving their antibacterial properties intact [64,75]. Polytetrafluoroethylene (PTFE) stands out among polymer options due to its high transparency, mechanical stability, flexibility, low toxicity, hydrophobicity, and anticorrosion properties [76,77]. Achieving desirable PTFE thin film thickness is possible via methods like sputtering, a chemical-free approach [76]. Following this approach, our group created transparent PTFE-coated zinc-doped silicon oxide (SZO/PTFE) films as smartphone panel coatings, offering antireflective and antibacterial properties [62]. Reactive co-sputtering formed 30 nm SZO films with controlled zinc doping, followed by 15 nm PTFE passivation through radio-frequency sputtering. Both the SZO and SZO/PTFE films had low refractive indices and high transmittance, much like SiO_2_ film and glass (Figure 4a). PTFE coating enhanced transmittance and hydrophobicity, enduring over time (Figure 4b,c). SZ0.29O/PTFE films achieved a wetting angle of ~105.2°, surpassing that of SZ0.29O films (WCA ~40°). Water dipping affected the nanostructures on SZO films but not SZO/PTFE films due to PTFE (Figure 4d,e). The nanostructures on SZO films impacted antibacterial effects (Figure 4f), while water-dipped SZO/PTFE films reduced both strains (Figure 4g). Water-dipped SZO/PTFE films had stronger antibacterial activity than SZO films. SZO/PTFE films are excellent for transparent, water-resistant, antibacterial displays due to their properties. 

**Figure 5:**
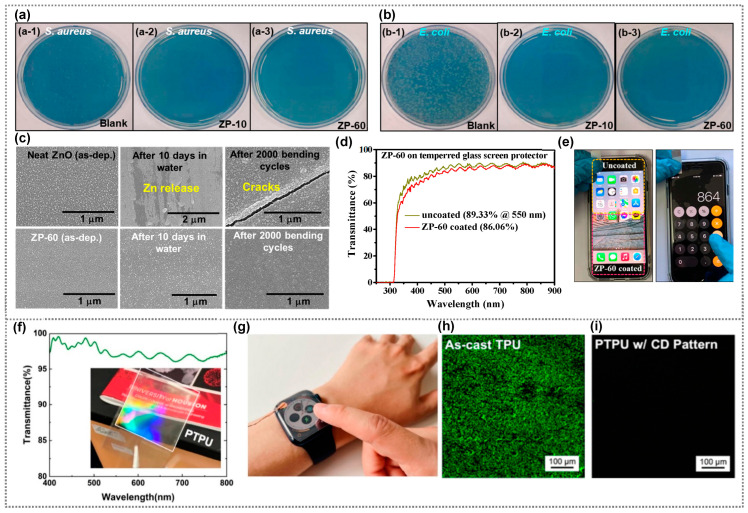
The antimicrobial activity of the various ZnO–PTFE composite coatings (ZP-10 and ZP-60) against (**a**) *S. aureus,* (**b**) *E. coli* after incubation for 24 h on (**a-1**, **b-1**) blank, (**a-2**, **b-2**) ZP-10, and (**a-3**, **b-3**) ZP-60 samples), Transmittance. (**c**) SEM images of as-deposited ZnO and ZP-60 films after 10 h dipping in DI water and 2000 bending cycles, (**d**) Transmittance of tempered screen protector with and without a coating of ZP-60, (**e**) ZP-60 coated-tempered glass screen protector attached to a smartphone. (**a**–**e**) Reprinted from Ref. [69]. Copyright 2022, Royal Society of Chemistry. (**f**) Transmittance of the transparent PTPU-TENG. (**g**) Captured picture of the transparent TENG serving as a screen protector for a smartwatch; confocal laser scanning microscopic images of (**h**) as-coated TPU, and (**i**) patterned TPU. (**f**–**i**) Reprinted from Ref. [80]. Copyright 2023, Elsevier.

In a study by Heo et al. [75], a protective layer (fluoride/SiO_2_ films) was added to silver nanoparticles (Ag NPs)/polyethylene terephthalate (PET) for improved transmittance, durability, and anti-fingerprint and antibacterial properties (Figure 4h). A 5 nm fluoride layer with fluorine and carbon enhanced anti-fingerprinting. Some 10 nm SiO_2_ buffered films were added for better adhesion. The 15 nm protective layer on even the Ag NPs maintained PET-like transmittance (~70% at 450 nm). It resisted fingerprints, stayed durable after swipes (Figure 4i), and retained antibacterial properties (Figure 4j). Recently, our group introduced co-sputtering to create antibacterial zinc oxide-polytetrafluoroethylene (ZnO–PTFE) composite coatings for displays [69]. These films with varying PTFE levels showcased a low refractive index, high transmittance, hydrophobicity, and potent antibacterial activity against *E. coli* and *S. aureus*. ZP-10 and ZP-60 samples, despite having differing zinc concentrations, achieved similar antimicrobial results (Figure 5a,b). This research also addressed zinc dissolution by combining ZnO with PTFE, ensuring lasting water stability and mechanical durability (Figure 5c). ZnO–PTFE films have emerged as potential antimicrobial coatings for touch devices, preserving transparency while curbing microbial contamination. Transparent ZnO–PTFE films’ practicality as a display coating was demonstrated on a tempered glass screen protector, with minimal transmittance drop (Figure 5d). The protector retained the smartphone screen’s color, brightness, and touch functionality, ensuring seamless touchscreen use without affecting display performance (Figure 5e).

Polyurethane (PU) stands as a promising polymer for wound care and antibacterial use, offering high absorptivity, stability, softness, flexibility, and low cytotoxicity in PU films/foams [78,79]. Chen et al. introduced a metal-free PTPU-TENG, which was transparent and constructed with TPU film on an ITO-PET substrate [80]. It exhibited high optical transparency and antibacterial properties through pattern replication. TENG’s transparency, verified by UV-vis spectrum analysis, maintained over 96% optical transmittance from 400 nm to 800 nm (Figure 5f). The pattern on PTPU acted as a diffraction grating, successfully replicating a master mold’s pattern. This eco-friendly, cost-effective antibacterial approach holds potential in wearables and hardware displays. A prototype of the transparent TENG integrated with a smartwatch screen demonstrated efficient power generation through finger tapping (Figure 5g). Addressing bacterial concerns on high-touch surfaces, the study evaluated PTPU-TENG’s long-term wearability against *E. coli* bacteria. Confocal laser scanning microscopy (CLSM) revealed limited bacterial attachment on TENG with a CD pattern due to optimized line width and groove depth (Figure 5h,i). CD pattern’s higher hydrophobicity and lower surface tension reduced cell adhesion and facilitated migration, showing PTPU-TENG’s potential for healthy, long-term wear with reduced bacterial interaction.

In the study by Canama et al. [70], some sugars were used in a composite with antibacterial materials to improve their performance. The authors successfully demonstrated an environmentally friendly method for antibacterial coating on mobile phone glass protectors using chitosan–silver nanoparticles (ChAgNPs). The nanocoating exhibited significant antibacterial activity against *E. coli* at 24 and 48 h, with a slight decrease in activity over time (from 49.80% to 32.60%). This approach shows great promise in creating effective and eco-friendly antibacterial coatings for mobile phone glass protectors. The antibacterial activity of thin films made from various materials against various microorganisms and their potential application as surface coatings are summarized in Table 2.

## 4. Antibacterial Polymer Composites for Sensor Applications

**Figure 6:**
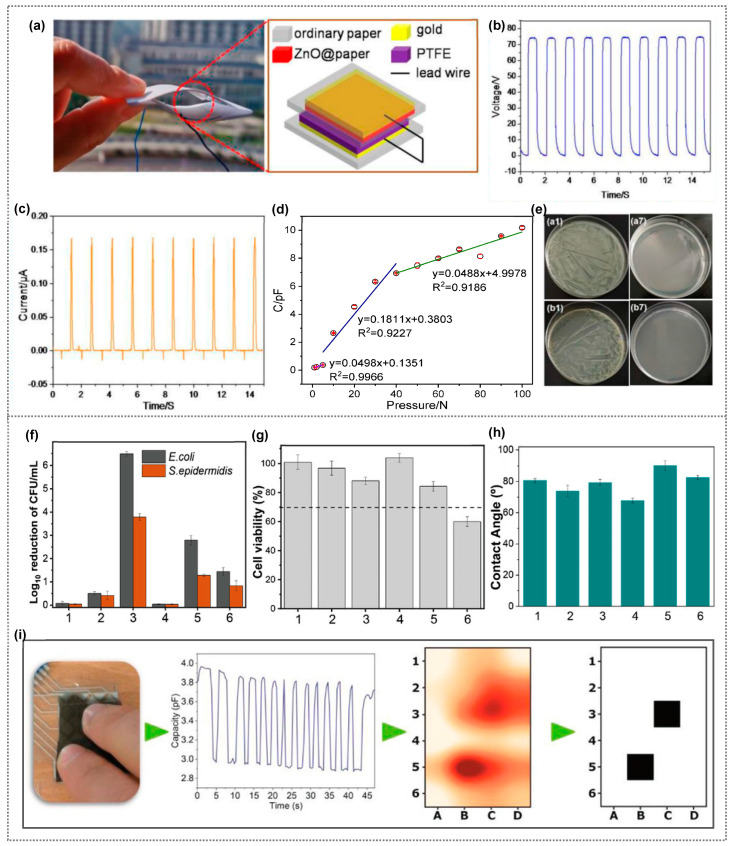
(**a**) Schematic structure of P-TENG with its captured image and corresponding (**b**) output voltage and (**c**) current of P-TENG; (**d**) Pressure sensitivity of P-TENG obtained from the maximum peak output amplitudes and (**e**) antibacterial activity of ZnO-paper composite against *E. coli* ((a1, a7) blank paper) and *S. aureus* ((b1, b7) ZnO@paper-5). (**a**–**e**) Reprinted from Ref. [94]. (**f**) Antibacterial activity, (**g**) cytotoxicity, (**h**) water contact angles of PVDF and PVDF composites with 0.5 and 5 wt.% of different fillers: (1) PVDF, (2) 05AgCu2/PVDF, (3) 5AgCu2/PVDF; (4) 05AgCu10/PVDF, (5) 5AgCu10/PVDF; (6) 5NiCu/PVDF. (**i**) Captured image of a 5AgCu2/PVDF capacitive touch sensor, along with the corresponding chart illustrating the touch locations of two fingers. (**f**–**i**) Reprinted from Ref. [95]. Copyright 2022, Wiley-VCH.

The rapid growth of Internet of Things (IoT) technology has fueled significant interest in real-time monitoring sensors for environments and human activities [81,82,83]. Sensors are vital for quantifying diverse external physical attributes, including light, pressure, temperature, chemicals, and biological properties [84,85]. Among these, nanogenerator-based self-powered sensors emerge as promising solutions, addressing traditional sensor limitations like power constraints and portability issues, shaping the future of sensor technology [86,87,88,89]. Nanogenerators harness renewable sources to generate electricity and are categorized as PENG (piezoelectric), TENG (triboelectric), and PyENG (pyroelectric) based on their operating principles. They serve as self-powered sensors, including pressure/strain, humidity, and temperature sensors [87]. By converting mechanical energy from vibrations, biomechanical movements, or waste heat into measurable electrical signals, they offer an innovative approach. Tactile sensors, frequently touched, can foster bacterial growth, posing health risks. Bacteria within the active material can even lower the electrical output of a nanogenerator, potentially impairing its ability to monitor human motion effectively [90]. Thus, incorporating antibacterial properties becomes vital for enhancing tactile sensors’ performance and preventing infections. Robust mechanical properties are essential for long-term operational stability in any sensor type. Polymer-based composites are vital for improving safety and the interactive experience. To this end, researchers have focused on developing antibacterial polymer composites [91,92,93]. By blending antibacterial materials with flexible polymers, they create innovative, effective solutions. Lin et al. developed ZnO@cellulose paper via cellulose fibers treated with an aqueous ZnCl_2_-urea eutectic, holding potential for paper-based TENG (P-TENG) as a friction layer (Figure 6a) [94]. P-TENG showcased an impressive max output voltage (V_OC_) of 77 V and a current (I_SC_) of 0.17 μA from external force (Figure 6b,c). A ZnO nanosheet in the cellulose paper enhanced P-TENG’s electrical output, combining triboelectric and electrostatic effects between ZnO@paper and PTFE. It served as a pressure sensor, generating capacitance, voltage, and current against external pressure. The observed sensitivities vary across tiny- (1–5 N), low- (5–40 N), and high-pressure (40–100 N) regions (Figure 6d), indicating P-TENG’s versatile pressure-sensing potential. Remarkably, it exhibits potent antibacterial activity against *E. coli* and *S. aureus*, suitable for wearables to prevent bacterial buildup during extended use. ZnO@paper effectively kills both Gram-positive (*S. aureus*) and Gram-negative (*E. coli*) bacteria (Figure 6e). This P-TENG with ZnO@paper suppresses and eliminates microorganisms during operation, meaning it is promising as an antibacterial solution for wearable devices. Costa et al. developed PVDF composites using metallic NWs with copper core and silver and nickel shell, exhibiting antimicrobial properties and high conductivity when embedded in PVDF [95]. In the PVDF matrix, NWs retained antimicrobial effects, reducing E. coli by 6.5 logs and S. epidermidis by 4.5 logs in 5AgCu2/PVDF composite (Figure 6f). Incorporating Ni-Cu (5NiCu/PVDF) showed cytotoxicity, possibly due to NWs leaching (Figure 6g). The samples demonstrated hydrophilic behavior in their water contact angle, indicating similar surface roughness between PVDF and composites (Figure 6h). Their work included a capacitive matrix using a 5AgCu2/PVDF composite with strong antimicrobial properties and a high dielectric constant, effectively detecting touch points (Figure 6i). Capacitive variation was about 24% (3.8 to 2.9 pF) between no finger touch and touch, showing non-cytotoxicity for certain filler contents and inhibiting bacterial growth when used as antimicrobial coatings on touch sensors. This device has potential for touch-sensitive applications and home automation, offering touch-sensing and antimicrobial features for high-traffic surfaces.

**Figure 7:**
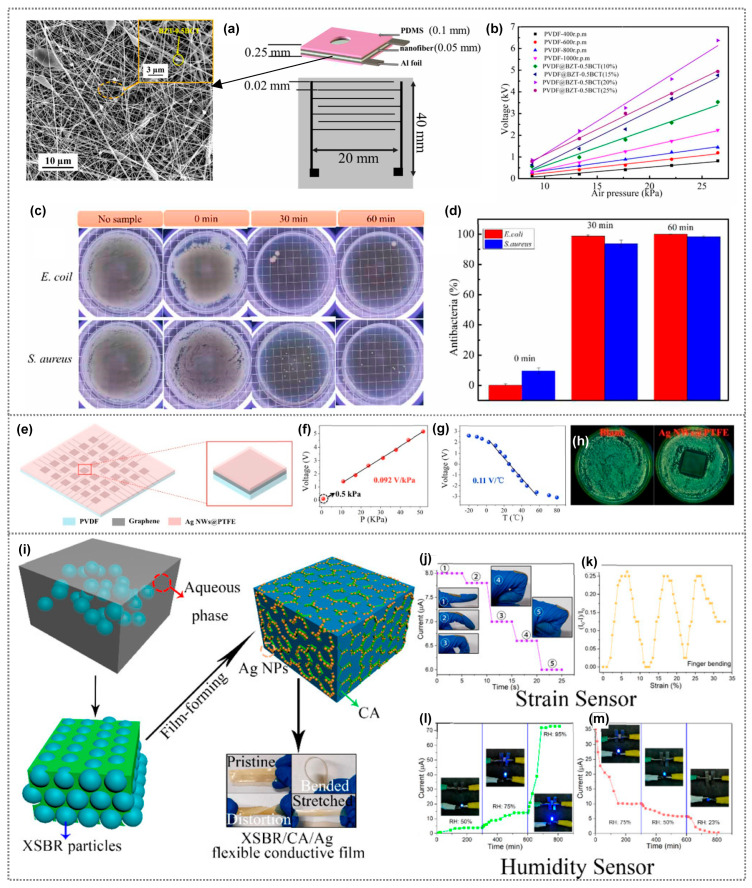
(**a**) PVDF/BZT−0.5BCT (20%) composite fibers and its schematic sensor. (**b**) Output voltages of sensors with respect to air pressure, (**c**) optical images and live bacterial counts of *E. coli* and *S. aureus* at 0 min, 30 min, and 60 min following ultrasonic treatment with PVDF/BZT−0.5BCT nanofibers, and (**d**) Antibacterial efficacy of BZT-0.5BCT/PVDF nanofibers against *E. coli* and *S. aureus*. (**a**–**d**) Reprinted from Ref. [96]. Copyright 2022, IOPscience. (**e**) Schematic representation of the self-powered antibacterial tactile sensor and its corresponding (**f**) pressure sensitivity and (**g**) temperature sensitivity. (**e**–**h**) Reprinted from Ref. [99]. Copyright 2023, Elsevier. (**i**) Structure of the flexible conductive XSBR/CA/Ag film, (**j**) change in the current of the sensor with increasing bending angle of the finger, and (**k**) change in (I_0_–I)/I_0_ with the bending angle of the finger. (**l**) Change in the current of the sensor with (**l**) increasing and (**m**) decreasing humidity. (**i**–**m**) Reprinted from Ref. [100]. Copyright 2020, American Chemical Society.

A research group developed a composite sensor for respiratory monitoring using high piezoelectric phase PVDF nanofibers created via electrospinning, enhanced with Ba(Ti_0.8_Zr_0.2_)O_3_–0.5(Ba_0.7_Ca_0.3_)TiO_3_ (BZT–0.5BCT) nanoparticles (Figure 7a) [96]. PVDF/BZT-0.5BCT (20%) composite fibers exhibited a 6.37 V output voltage, a 0.24 V/Kpa sensitivity, a fast response, durability, and a 2.50 mg detection limit (Figure 7b). They also demonstrated potent antibacterial properties against *E. coli* and *S. aureus* (Figure 7c). After 60 min of ultrasonic vibration, nearly complete bacteria elimination occurred, with 99.86% and 98.39% antibacterial rates for *E. coli* and *S. aureus*, respectively (Figure 7d). BZT-0.5BCT nanoparticles produced a piezoelectric potential (E) under ultrasound, generating reactive oxygen species (ROS) and damaging bacterial cells. These nanofibers offer active sterilization, surpassing traditional masks in protection. Li et al. developed a novel stretchable Ag/MXene-Poly(vinyl alcohol) hydrogel with remarkable properties [97]. This hydrogel exhibited strong antibacterial activity comparable to ampicillin, particularly at 1 wt% Ag/MXene concentration, eradicating over 93% of S. aureus and 95% of *E. coli*. It also served as a wearable skin-like sensor, showing excellent strain sensitivity (GF of 2.11 within 0–100% strain range), becoming even more sensitive (GF of about 3.26) at higher strains (100–500%). Wang et al. introduced a breathable, biodegradable, and antibacterial e-skin using an all-nanofiber triboelectric nanogenerator [98]. Silver nanowires (Ag NWs) were incorporated between polylactic-co-glycolic acid (PLGA) and polyvinyl alcohol (PVA), creating a hierarchical porous structure for enhanced contact electrification and moisture transfer. The e-skin displayed tailored antibacterial and biodegradable properties by adjusting Ag NW concentration and material selection. The e-skin exhibited strong antibacterial effects against *E. coli* and *S. aureus*, with up to 88% reduction in surviving bacteria. The mechanism involves silver ions disrupting cell membranes and interacting with cellular proteins. Moreover, the e-skin responded linearly up to 40 kPa pressure, making it suitable for real-time, self-powered monitoring of physiological signals and movements. This work presents a promising step towards multifunctional e-skins.

## 5. Antibacterial Polymer Composites for Multifunctional Applications

In more recent years, the demand for skin-like multifunctional sensors capable of sensing multiple stimuli is increasing. However, integrating multiple sensors with different functions often requires complex fabrication processes. With this goal, Ma et al. developed a self-powered flexible antibacterial tactile sensor featuring a sandwich structure with an Ag nanowires@PTFE polymer (Ag NWs@PTFE) film, graphene electrode, and polarized PVDF film, utilizing a triboelectric-piezoelectric-pyroelectric multi-effect coupling mechanism for multiple signals sensing in a pixel unit (Figure 7e) [99]. It has high sensitivity for pressure and temperature monitoring without requiring external power. The tactile sensor exhibits a pressure sensitivity of 0.092 V/KPa with a response time of approximately 130 ms (Figure 7f). The sensor demonstrates a linear response, allowing it to function as a precise temperature sensor with a sensitivity of 0.11 V/°C. This capability makes it well-equipped to accurately detect and measure changes in temperature (Figure 7g). Additionally, the Ag NWs@PTFE-based sensor shows strong antibacterial properties, effectively inhibiting the growth of *E. coli* (Figure 7h). This is due to the reactivity of nanoscale Ag, which causes structural changes in the bacterial cell wall and membrane, leading to cell distortion and death. The novel sensor design provides diverse and practical applications in multifunctional domains. Xu et al., described a method to fabricate conductive stretchable films with antibacterial properties and good mechanical strength [100]. The conductive film, composed of carboxylic styrene-butadiene rubber (XSBR), citric acid (CA), and silver nitrate (AgNO_3_), has prominent potential applications in wearable sensors for monitoring finger movements, and as a humidity sensor (Figure 7i). A simple strain sensor design based on XSBR/CA-20/Ag conductive film effectively tracks finger joint motion, showing rapid response (~1 s) and stability during static periods. The film’s unique features during continuous finger bending make it a promising material for wearable strain sensors. Mold-pressing further improves its behavior, enhancing its competitiveness for such applications (Figure 7j,k). Additionally, the conductive film’s sensitivity to humidity, attributed to the hygroscopic nature of CA, enables its potential use as a humidity sensor (Figure 7l,m). Moreover, AgNP flexible conductive films demonstrate remarkable antibacterial activity against both *E. coli* and *S. aureus*, making them promising materials for flexible antibacterial applications and the real-time monitoring of human body movements.

**Figure 8:**
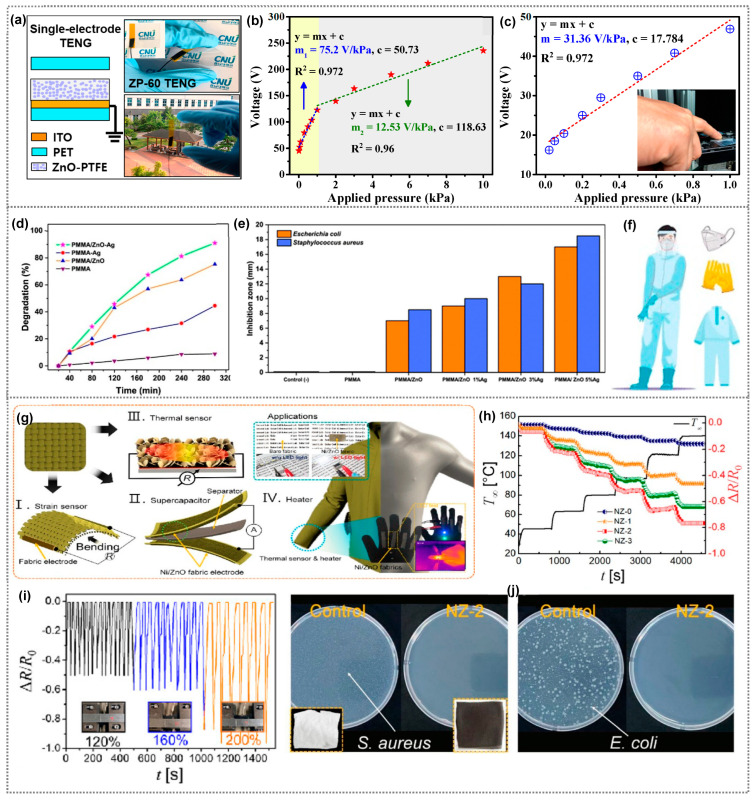
(**a**) A schematic illustration of the single-electrode TENG structure, accompanied by digital photographs and its corresponding (**b**) pressure sensitivity and (**c**) pressure sensitivity. (**a**–**c**) Reprinted from Ref. [69]. Copyright 2022, Royal Society of Chemistry. (**d**) Percentage of photocatalytic degradation of MB on pure PMMA, PMMA/Ag NF, PMMA/ZnO, and PMMA/ZnO–Ag NF Mats, (**e**) antibacterial activity of PMMA, PMMA/ZnO and PMMA/ZnO NF Mats with different concentrations of Ag%, and (**f**) a schematic depiction of the incorporation of PMMA/ZnO–Ag NF mats in protective clothing. (**d**–**f**) Reprinted from Ref. [101]. Copyright 2021, American Chemical Society. (**g**) Schematic representation showcasing the multifunctional capabilities of Ni/ZnO fabric as a strain sensor, thermal sensor, and supercapacitor, and (**h**) Variation in the ΔR/R_0_ of the Ni/ZnO fabric specimens in response to ambient temperature. (**i**) Change in ΔR/R_0_ with respect to various strain percentages, and (**j**) the antibacterial activity of Ni/ZnO fabric specimens against S. aureus and *E. coli*. (**g**–**j**) Reprinted from Ref. [102]. Copyright 2022, Elsevier.

Our group reported a TENG-based antimicrobial self-powered touch sensor utilizing ZnO–PTFE composite films (Figure 8a) [69]. The ZnO–PTFE composite films developed also exhibit multifunctional abilities, including antibacterial properties against E. coli and S. aureus bacteria, as discussed earlier in the previous section. The ZnO–PTFE composite-based TENG shows impressive performance, achieving a high triboelectric output voltage of 224 V, a current density of 21.4 μA/cm^2^, and a power density of 1.65 mW/cm^2^. The TENG demonstrates outstanding pressure-sensing ability, exhibiting a sensitivity of 75.31 V/kPa and touch sensitivity of 31.36 V/kPa (Figure 8b and Figure 8c, respectively) within the pressure ranges of 0.01 to 1.0 kPa. Additionally, it boasts good mechanical durability and long-term operational stability. The method used for creating these smart antimicrobial coatings enables them to possess diverse functionalities, making them a suitable choice for enhancing user safety in various interactive devices. The study reported by Karagoz et al. investigated the use of multifunctional electrospun PMMA nanofibers decorated with ZnO nanorods and Ag nanoparticles (PMMA/ZnO−Ag NFs) for protective mats [101]. These nanofibers were prepared on a nonwoven fabric using a straightforward electrospinning method. The material showcased four key functionalities: antibacterial and antiviral properties, photocatalytic ability for organic pollutant degradation, and reusability as a surface-enhanced Raman scattering substrate for trace pollutant analysis. The composted nanofibers demonstrated the highest photodegradation efficiency for MB dye, degrading 91% of the dye after 300 min compared with pure materials (Figure 8d). They prompt us to speculate that the PMMA/ZnO-Ag nanofibers inhibit both *E. coli* and *S. aureus* bacteria. *S. aureus* was more susceptible, showing a strong inhibition zone of around 18.5 mm (Figure 8e). The researchers suggest that this versatile material holds great potential for protective clothing applications, offering combined passive and active protection along with the ability to sense and analyze pollutants (Figure 8f). Similarly, the fabric developed by Kim et al. displayed multifunctional properties including sensing, heating, and supercapacitive properties (Figure 8g) [102]. Further, these fabrics are wearable, stretchable, washable, hydrophobic, and processes antibacterial activity, due to which they have applications in healthcare monitoring, smart sportswear, and energy storage. These fabrics were created by electroplating nickel nanocones and zinc oxide nanosheets onto them. They suggest that the integration of nickel enhances electrical conductivity, while ZnO provides pseudocapacitance. The fabric’s resistance changes with temperature and strain, and it generates heat when voltage is applied. The fabric’s resistance changes by 80% at T_∞_ = 140 °C (Figure 8h), and nearly 100% when stretched to 200% of its length (Figure 8i). Further, strong antibacterial performance was achieved by Ni/ZnO fabric, eradicating bacteria completely by disrupting their metabolism with Ni ions’ redox reactions (Figure 8j), making it suitable for various industries’ portable and wearable electronic textiles. 

**Figure 9:**
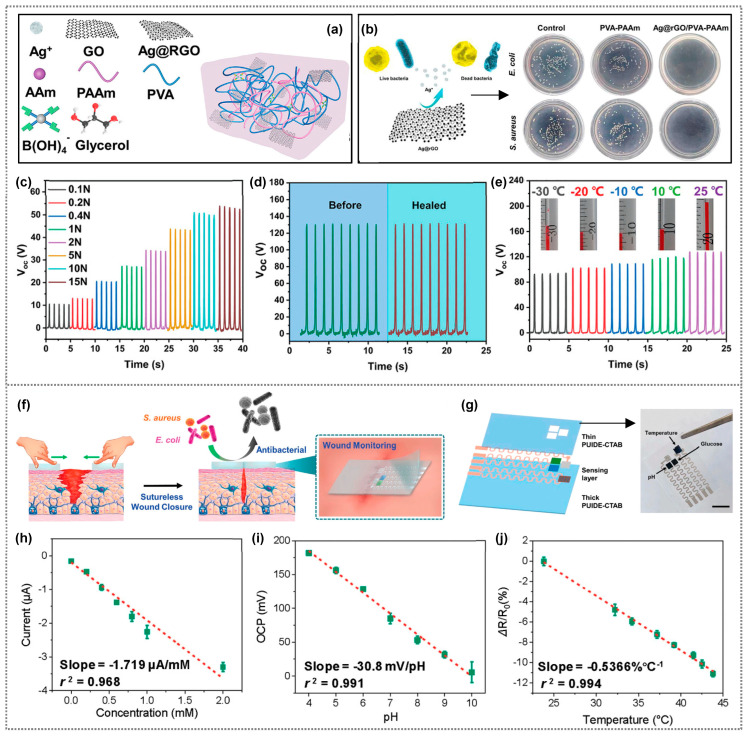
(**a**) Schematic of Ag@rGO/PVA-PAAm organohydrogel. (**b**) Schematic diagram illustrating the antibacterial properties of the organohydrogel, along with captured photographs showing the distribution of *E. coli* and *S. aureus* colonies after co-culture with different materials. (**c**) Open-circuit voltage of the O-TENG under different forces. (**d**) Open-circuit voltage (Voc) of the O-TENG before damage and after self-healing. (**e**) Open-circuit voltage of the O-TENG at different temperatures (−30 to 25 °C). (**a**–**e**) Reprinted from Ref. [103]. Copyright 2022, Wiley-VCH. (**f**) Schematic representation of MFWD for wound closure, antibacterial activity, and infection monitoring. (**g**) Schematic diagram and actual photograph of the sensing component of MFDW featuring three distinct sensors: glucose (PB/GOx), pH (PANI), and temperature (PEI/rGO). Scale bar: 1 cm. (**h**) Glucose calibration: linear 0–200 × 10^−6^ m, sensitivity 1.72 μA/mM, r^2^ = 0.968. (**i**) pH sensing: linear, sensitivity −30.8 mV/pH, r^2^ = 0.991. (**j**) Temperature sensing: linear, sensitivity ≈0.54%/°C, r^2^ = 0.994. (**f**–**j**) Reprinted from Ref. [104]. Copyright 2022, Wiley-VCH.

On the other hand, another research group produced an innovative organohydrogel-based triboelectric nanogenerator (O-TENG) with multiple functionalities, including antibacterial, anti-freezing, stretchable, and self-healing properties [103]. Their O-TENG utilizes Ag nanoparticles on reduced graphene oxide sheets (Ag@rGO) integrated into a dual-network organohydrogel (Ag@rGO/PVA-PAAm) with dynamic borate bonds (Figure 9a). The organohydrogel exhibits high conductivity, good stretchability, and resistance to freezing. When used as the electrode layer of the O-TENG, Ag@rGO/PVA-PAAm effectively inhibits the Gram-negative bacterium *E. coli* and Gram-positive bacterium *S. aureus,* while remaining highly compatible with cells (Figure 9b). The O-TENG sensor harvests mechanical energy and showed high sensitivity to tiny forces, reaching 10 V, 0.15 μA, and 3 nC for 0.1 N (Figure 9c), and the sensitivity reached 18.34 V/N for forces below 1 N. Further, the hydrogel processes’ self-healing ability maintains the TENG’s electrical output after it is damaged and self-repaired, similar to an intact electrode (Figure 9d). The O-TENG demonstrates stable output performance at both room temperature and low temperatures (as low as −30 °C) due to its self-healing and anti-freezing properties (Figure 9e).

Tang et al., reported a novel multifunctional wound dressing (MFWD), which is a self-healing, antibacterial wound dressing for sutureless wound closure (Figure 9f) [104]. A biocompatible elastomer (PUIDE) was synthesized via the polycondensation of hydroxyl-terminated polybutadiene (HTPB), 1,10-decanediol (DE), and isophorone diisocyanate (IPDI), catalyzed by dibutyltin dilaurate (DBTDL). Cetyltrimethylammonium bromide (CTAB) (1% mass ratio to PUIDE) was added to form a homogeneous mixture, poured into a Teflon mold, and dried in a vacuum oven to obtain PUIDE-CTAB elastomer without bubbles. It has high toughness, biocompatibility, and inhibits bacterial growth. MFWD’s sensing part comprises three layers: a thick PUIDE-CTAB for sutureless wound closure, a sensing layer (glucose, pH, temperature), and a thin PUIDE-CTAB to protect the electrode from contacting the wound bed. A serpentine electrode was fabricated using spray-coated silver nanowires (AgNWs) (Figure 9g). In vivo tests show it aids wound closure and healing, with integrated sensors for real-time monitoring of temperature, pH, and glucose levels. The glucose sensor showed excellent linearity and a sensitivity coefficient (SC) of ~1.72 μA/mM with a regression coefficient of 0.968 in the concentration range typical of wound milieu (Figure 9h). It exhibited good repeatability in successive tests. The pH sensor was calibrated and showed excellent linear response, high sensitivity (with SC of −30.8 mV/pH), a regression coefficient of 0.991, and reliability for monitoring pH variations at wound sites (Figure 9i). As a temperature sensor, it responded rapidly and reliably to temperature variations (23.8 to 43.8 °C) with excellent linearity (SC −0.537%/°C), r^2^ = 0.994) (Figure 9j). The sensor showed reliable repeatability, and its dynamic response was fast and suitable for monitoring wound temperature (35.8 to 42.6 °C). PUIDE-CTAB has the potential to damage the membrane integrity of both *E. coli* and *S. aureus*. Indeed, all these studies unequivocally showcase the promising potential of antibacterial polymer composites for multifunctional applications, encompassing the sensing of multiple physical signals and energy harvesting, among other elements.

## 6. Conclusions and Future Perspectives

Antibacterial polymer composite materials offer a host of advantages that make them highly attractive for various applications. First and foremost, these materials possess inherent antibacterial activity, effectively inhibiting bacterial growth and mitigating infection risks. Moreover, many of these composites are biocompatible, rendering them safe for medical and healthcare purposes without causing harm to living tissues. Additionally, some antibacterial composites exhibit self-healing properties, enhancing their durability and longevity. Their multifunctional nature enables them to serve multiple purposes, such as wound healing, sensing, and energy harvesting, making them versatile for diverse applications. Furthermore, these composites may be environmentally friendly, being derived from eco-friendly materials, thus contributing to sustainable practices. Their cost-effectiveness adds to their appeal, making them economically viable for various industries. Improved material properties, including enhanced mechanical strength and flexibility, further boost their suitability for diverse applications. With long-lasting antibacterial effects and ease of fabrication using conventional techniques, antibacterial polymer composites represent a promising solution for numerous industries, from healthcare to electronics and beyond. Antibacterial polymer composite materials offer valuable advantages in both display coatings and sensors. In display coatings, they provide bacterial protection, enhance durability, and reduce cross-contamination, promoting a cleaner and safer environment. These coatings maintain the display’s visual clarity and require less maintenance. In sensors, antibacterial materials ensure reliable data by preventing contamination, leading to more accurate and consistent measurements. They also prolong the sensor lifespan and improve precision, making them particularly beneficial in medical and healthcare applications. Overall, these materials contribute to better hygiene, durability, and accuracy in both display coatings and sensors. Although antibacterial polymer composites offer targeted protection against bacteria, they have limitations. Their narrow spectrum of activity, potential for bacterial resistance, environmental concerns, and toxicity raise issues. Their short-term effectiveness, high production costs, and regulatory approval can also be problematic. These composites may compromise mechanical properties and lose antibacterial activity over time. Ongoing research seeks to improve their effectiveness, safety, and sustainability, addressing these challenges in various applications.

The future of antibacterial polymer composite materials in display coatings and sensors is promising, and they hold great potential for various applications. In the realm of display coatings, researchers are focused on developing advanced antimicrobial formulations capable of combating a wider range of pathogens, including drug-resistant bacteria. Efforts are being made to design coatings with long-lasting antibacterial effectiveness, ensuring sustained protection against microbial contamination over extended periods. Additionally, there is growing interest in integrating self-cleaning capabilities into display coatings, reducing the need for frequent maintenance and enhancing overall hygiene. Moreover, the emergence of smart antimicrobial coatings is anticipated, where coatings can actively sense bacterial contamination, and trigger the release of antimicrobial agents in response, in order to ensure continuous protection. In sensors, the potential of antibacterial polymer composites is equally promising. Scientists are exploring the integration of antibacterial materials into biocompatible and implantable sensors, enabling continuous monitoring within the human body for healthcare and medical applications. The future will witness the seamless integration of antibacterial sensors with the Internet of Things (IoT) and wearable devices, facilitating real-time monitoring of environmental and physiological parameters for various applications. Moreover, researchers are working towards creating multi-functional sensors that combine antibacterial properties with other features such as self-healing capabilities, energy harvesting, and responsiveness to specific stimuli, thereby broadening their range of applications and utility. Lastly, the focus is on developing smaller and more efficient antibacterial sensors that consume minimal power, ensuring long-lasting and unobtrusive monitoring across diverse industries. However, integrating antibacterial polymer composites into displays and sensors faces key challenges. These encompass preserving antibacterial effectiveness while ensuring durability and functionality, as well as compatibility with sensors and display clarity. Safety concerns and regulatory compliance are essential for handling antibacterial agents, while cost and adapting to evolving pathogens are economic and efficacy factors. Balancing optical and mechanical properties, addressing user acceptance, and achieving scalability are also crucial for successful integration. Tackling these challenges is vital for maximizing the benefits of antibacterial materials in display and sensor applications.

## Figures and Tables

**Table 1:** Comparison of the antibacterial activity of polymer composite materials against various microorganisms.

Composite Material	Bacteria/Virus/Fungi	Antibacterial Activity/Reduction	Ref.
Q-DMHC48/Ag NPs	*S. aureus*, *E. coli*, *P. aeruginosa*, *K. pneumoniae*, *Candida* spp., *Cryptococcus* spp.	>99.99% (*S. aureus*) 100% (*E. coli*)	[29]
WPUL/Ag	*S. aureus*, *P. aeruginosa*	99.99%, 99.99%,	[32]
PBAT/Cu, PBAT/Cu|Cu_2_O, PBAT/CuSO_4_	*S. aureus*, *A. baumanni*, *E. faecalis*, *S. mutan*	-	[40]
Cellulose/Cu nanofillers	*S. aureus*, *K. pneumoniae*	-	[34]
Polyurethane/Cu	*S. aureus*, *E. coli*	2 Log10 < cell density < 3 Log10, ≥3 Log10	[35]
Polypropylene/Cu NPs	*S. aureus*, *P. aeruginosa*	100%	[36]
Chitosan/Au NPs	*S. aureus*, *P. aeruginosa*	-	[38]
Ag-Au/PTFE	*S. aureus*, *S. epidermidis*	-	[39]
PMMA/ZnO	*E. coli*	1 Log CFU/ml	[45]
PLGA/CuO NFs	*S. aureus*, *E. coli*	-	[47]
Chitosan/NiO-MgO	*S. aureus*, *E. coli*	98%, 92.3%	[48]
Cationic Polymers-GO and GR	*S. aureus*, *E. coli*, *S. epidermidis*, *P. aeruginosa*	-	[54]
Polyvinyl N-carbazole/SWNT	*E. coli*, *B. subtilis*	94%, 90%	[56]
Polyvinyl N-carbazole/GO	*E. coli*	90%	[53]

**Table 2:** Antibacterial activity of various material-based thin films against different microorganisms and their potential applications as surface coatings.

Material	Microorganism	Antibacterial Activity/Reduction	Application	Ref.
Zn doped SiO_2_/PTFE	*S. aureus*, *E. coli*	99.99748%, 99.999947%	Smartphone panel	[62]
ZnO-PTFE	*S. aureus*, *E. coli*	4.7, 6.2	Displays	[69]
Chitosan-silver nanoparticles	*E. coli*	49.8%	Mobile phone glass protectors	[70]
ZnO	*S. aureus*, *E. coli*	99.99999%, 99.99668%	Smartphone panel	[71]
ZnAl_2_O_4_	*S. aureus*, *E. coli*	4.5, 6.3	Surface coating	[74]
SiO_2_-PTFE-coated Ag NPs	*S. aureus*, *E. coli*	4.4, 6.4	Anti-fingerprint hydrophobic coating	[75]
Nano pattern-TPU	*E. coli*	>99%	Touch sensor, Displays	[80]

## Data Availability

Data sharing is not applicable.
